# Do everyday problems of people with chronic illness interfere with their disease management?

**DOI:** 10.1186/s12889-015-2303-3

**Published:** 2015-10-01

**Authors:** Lieke van Houtum, Mieke Rijken, Peter Groenewegen

**Affiliations:** Netherlands Institute for Health Services Research (NIVEL), P.O. Box 1568, 3500 BN Utrecht, Netherlands; Department of Sociology and Department of Human Geography, Utrecht University, P.O. Box 80140, 3508 TC Utrecht, Netherlands

**Keywords:** Self-management, Chronic illness, Life context, Everyday problems

## Abstract

**Background:**

Being chronically ill is a continuous process of balancing the demands of the illness and the demands of everyday life. Understanding how everyday life affects self-management might help to provide better professional support. However, little attention has been paid to the influence of everyday life on self-management. The purpose of this study is to examine to what extent problems in everyday life interfere with the self-management behaviour of people with chronic illness, i.e. their ability to manage their illness.

**Methods:**

To estimate the effects of having everyday problems on self-management, cross-sectional linear regression analyses with propensity score matching were conducted. Data was used from 1731 patients with chronic disease(s) who participated in a nationwide Dutch panel-study.

**Results:**

One third of people with chronic illness encounter basic (e.g. financial, housing, employment) or social (e.g. partner, children, sexual or leisure) problems in their daily life. Younger people, people with poor health and people with physical limitations are more likely to have everyday problems. Experiencing basic problems is related to less active coping behaviour, while experiencing social problems is related to lower levels of symptom management and less active coping behaviour.

**Discussion:**

The extent of everyday problems interfering with self-management of people with chronic illness depends on the type of everyday problems encountered, as well as on the type of self-management activities at stake.

**Conclusions:**

Healthcare providers should pay attention to the life context of people with chronic illness during consultations, as patients’ ability to manage their illness is related to it.

## Background

Self-management of (chronic) illness by patients has been promoted by healthcare providers and policymakers in many Western societies as a cornerstone of modern healthcare [1]. Self-management requires people with chronic illness to monitor their health status, take medication as prescribed, interact with healthcare providers and manage the impact of the illness on physical, psychological and social functioning [2]. However, people with chronic illness often find it difficult to perform effective self-management [3], as indicated by e.g. low rates of medication adherence [4, 5], poor levels of disease control [6], and the modest positive effects of self-management interventions [7, 8].

Previous studies have shown that people with chronic illness experience tension between managing and controlling their chronic illness while being able to do what they would like to do with their lives [9–11]. As Corbin and Strauss state (1985), the ideal context for self-management would be a controlled environment in which influences of everyday life are minimised [12]. However, in reality, people with chronic illness need to consistently balance the demands of the illness against those of everyday life, as the lives of people do not solely consist of taking care of their chronic illness. People with chronic illness have jobs, partners, children, friends, and hobbies, and experience the delights and concerns that come with them. Moreover, due to their illness they may encounter additional problems in daily life, for instance problems related to living independently (e.g. housing, finances). These problems might be partly the consequence of having a chronic illness, but they might also influence the way people manage their illness.

The Social Production Function theory of Lindenberg and colleagues [13–15] states that people produce well-being by achieving goals, within the set of resources and constraints they face. Based on this theory, we argue that people with chronic illness need to prioritise their goals and decide where their resources such as time, energy, money and social support will go. Facing, for instance, financial, marital or housing problems, people may prioritise coping with these problems as more important than managing their chronic illness. Solving everyday problems requires resources, which can then no longer be used to manage the chronic illness. For example, research of Townsend et al. (2006) indicated that people with chronic illness sometimes gave priority to maintaining a ‘normal’ life at the expense of controlling symptoms [11].

“The process of self-management could be eased if the particular circumstances and the broader context in which it takes place are addressed by practitioners” [11]. Understanding how everyday life affects self-management might help to provide better support. Most research, however, focuses on how chronic illness complicates maintaining a normal life. Until now, little attention has been paid to the opposite, namely how everyday life influences the level of self-management of people with a chronic illness. Therefore, the purpose of this study is to examine to what extent problems in everyday life intervene with the level of self-management of people with chronic illness.

### Hypotheses

To guide our research, we formulated the following hypotheses:

1. Recognising and managing symptoms of a chronic illness requires time and energy. Examples of symptom management are monitoring of glucose level or blood pressure when you have diabetes or cardiovascular diseases, controlling shortness of breath when you have asthma or COPD, or doing exercises to maintain flexible when you have arthritis. We expect that symptom management will be neglected when people have everyday problems, such as financial, work-related, marital, or social problems, that also require their attention. We therefore hypothesise that *experiencing problems in everyday life will be negatively associated with the level of daily symptom management of people with chronic illness*.
2. Being actively involved in the treatment of the illness by adhering to treatment regimens, visiting healthcare providers and participating in decision-making will also require time and energy from people with chronic illness. However, these self-management tasks are more likely to be performed within a medical context, in close collaboration with healthcare professionals. Therefore, we expect that the effect of everyday problems on patients’ active involvement in the treatment will be limited. In effect, we hypothesise that *experiencing problems in everyday life will be negatively associated with the level of active involvement in treatment of people with chronic illness, but to a lesser extent than their symptom monitoring and management*.
3. Dealing with the consequences of having a chronic illness on physical, emotional and social wellbeing (coping) may be particularly complicated when people also have other problems. We expect that this aspect of self-management will be influenced most, as there are many similarities between having to deal with everyday problems and coping with chronic illness. We therefore hypothesise that *experiencing problems in everyday life will be negatively associated with the coping behaviour of people with chronic illness*.

## Methods

### Study sample

The sample of the present study consisted of members of the National Panel of people with Chronic illness or Disability (NPCD), a nationwide prospective panel-study on the consequences of chronic illness in the Netherlands [16]. For this study, we only included the participants with chronic illness. Participants with chronic illness were recruited from more than a hundred general practices (random samples of general practices drawn from the Dutch registration of General Practices [17]). These panel members were selected according to the following criteria: diagnosed with a somatic chronic disease by a certified medical practitioner, aged ≥15, not permanently institutionalised, aware of the diagnosis, not terminally ill (life expectancy >6 months according to the general practitioner), mentally capable to participate, and sufficiently proficient in Dutch. Potential panel members received an information letter about the panel and were asked to fill in a reply form whether or not they want to join the panel. If they were interested, they received a questionnaire on their demographic characteristics. When that questionnaire was returned, they were considered members of the panel. Annually, 500 new panel members were selected via the standardised procedure to replace panel members who withdrew or who had participated for the maximum term of 4 years. NPCD is registered with the Dutch Data Protection Authority; all data were collected and handled in accordance with the privacy protection guidelines of the Authority.

At inclusion, NPCD participants received a questionnaire on their socio-demographic characteristics. In addition, general practitioners (GP) provided (with patients’ permission) medical information about the panel members (chronic diseases diagnosed, dates of diagnoses, health status etc.). In April 2013, a questionnaire about self-management, everyday life problems and perceived general health was sent to the panel members. A total of 1731 patients diagnosed with a chronic disease completed this questionnaire (response = 80 %).

### Operationalisation

#### Self-management

We used the Dutch version of the Partners in Health Scale (PIH-Dutch) to measure patients’ self-management knowledge and behaviour. This scale was originally developed as part of the ‘Flinders Program of Chronic Care Self-Management [18, 19]. The PIH-Dutch scale consists of 12 items, which were answered on a scale ranging from 0 ‘low self-management’ to 8 ‘high self-management’. Examples of items are: ‘I have the ability to take action when my symptoms get worse’, ‘I have the ability to arrange appointments as recommended by my healthcare provider’ and ‘I have the ability to manage the impact of the illness on my social life’. As the first answering options of the original scale were all at a very close distance from each other resulting in a distribution very skewed to the right, we recoded the lower scores (0–3 = 0, 4–5 = 1, 6 = 2, 7 = 3 and 8 = 4). Four components of self-management were distinguished, namely knowledge (two items; knowledge about illness and treatment), recognition and management of symptoms (two items; monitor symptoms and act when symptoms worsen), active involvement in treatment (four items; taking medications as prescribed, attend appointments, shared decision-making) and coping with consequences (four items; dealing with effects on physical, emotional and social wellbeing and progress towards healthy life). As we focused in this study solely on self-management behaviour, we did not include the knowledge scale. Scale scores were computed by dividing the sum of respondents’ item scores by the number of items filled in, and range from 0 to 4, with higher scores indicating better self-management.

#### Everyday problems

To assess everyday problems we used the biographical list of problems (BIOPRO), developed by Hosman [20]. In this questionnaire, respondents were asked to indicate whether or not they have recently (no specific time frame given) encountered any of the following problems: financial, housing, employment, with partner, with children, with other people, sexual, with leisure time. Based on an exploratory factor analysis using principal component extraction with varimax rotation, we distinguished two types of problems, namely problems related to basic needs (financial, housing, employment) and problems related to social needs (partner, children, other people, sexual, leisure time). Based on this distinction, we constructed two dichotomous variables: basic problems and social problems (both scored into 0 ‘having no problems’ and 1 ‘having problems’).

#### Socio-demographic, illness and health status characteristics

In our study, we included the following socio-demographic characteristics of the participants: age, gender and highest level of education, classified as low (primary education, lower secondary and lower vocational education), intermediate (intermediate secondary and intermediate vocational education) and high (higher vocational education and university). We included these socio-demographic characteristics as we expected that these characteristics would have an effect on having everyday problems as well as self-management behaviour.

In addition, we used data provided by their GPs: type of chronic disease(s) diagnosed (coded by means of the International Classification of Primary Care [21]) and presence of more than one chronic disease (multimorbidity). Patients’ self-rated general health was measured by the general health scale of the RAND-36 Short Health Status Survey, ranging from 1 ‘poor health’ to 100 ‘excellent health’ [22]. Finally, the severity of physical limitations was assessed by the SCP physical disability indicator [23], a self-report questionnaire distinguishing four levels: none, mild, moderate and severe. This indicator assessed people’s ability to perform different tasks and activities, such as the ability to walk for short period of time, walk for a longer period of time, do odd jobs around the house, read the newspaper, hear what is being said during conversations, etc.

### Analyses

Descriptive analyses were performed to provide information about the characteristics of the study sample and to describe the everyday problems people with chronic illness encounter. To assess whether having basic or social everyday problems was related to the socio-demographic, illness and health status characteristics of people with chronic illness, we performed two multivariate logistic regression analyses (one with basic problems as dependent variable and the other with social problems as the dependent variable).

Next, we assessed the relationship between having basic or social problems and level of self-management. The problem in assessing this relationship is that having basic or social problems is not exogenous. Personal and health characteristics are related to self-management and to having problems. This makes it difficult to estimate the relationship of having problems and self-management. In other words, there are confounding variables that might influence both the outcomes (in our study, level of self-management of people with a chronic illness) and comparison groups (people having everyday problems versus those not having everyday problems). We used propensity score matching (PSM) to solve this problem as much as possible. The propensity score is a balancing score: conditional on the propensity score, the distribution of observed covariates will be similar between chronically ill people with and without everyday problems [24, 25]. Models were adjusted for age, sex, education, comorbidity, perceived general health and physical limitations and we inspected the diagnostics for propensity score analysis (checking for balance in the covariates). PSM is one way of approaching the problem and has its own assumptions that are not perfectly met in our study. Our assumption was that given similar background characteristics having everyday problems (the ‘treatment’) or not (the ‘controls’) could be considered as randomly assigned. Therefore, we also conducted multivariate linear regression analyses as a sensitivity analysis of our findings in the propensity score matching. In addition, as we could not include an interaction effect in the PSM analysis between having basic and social problems on the level of self-management, we also conducted a multivariate linear regression analyses in which we included this interaction effect as well.

The panel members were originally selected from general practices, resulting in a hierarchical data structure. Since intra-class correlations showed hardly any clustering of self-management behaviour within general practices (mean 0.01), and the likelihood ratio test did not show that multilevel analyses had an advantage over ordinary regression analyses, single-level regression analyses were conducted. All analyses were performed using Stata 13.0.

## Results

### Description study sample

The mean age of the study sample was 61.8 years (SD 14.3) and 54 % of the respondents were female. Cardiovascular disease (26 %), COPD (22 %) and asthma (20 %) were the most common chronic diseases within the sample. Half of the study sample (48 %) was diagnosed with more than one chronic (somatic) disease. The mean perceived health score of the study sample was 52.8, which is substantially lower than the mean score found in general population samples [26]. Forty-one percent of the respondents had no physical limitations, 29 % mild limitations, 22 % moderate and 8 % severe limitations.

### Problems in everyday life

A third (37 %) of the respondents reported recently experiencing one or more problems in their everyday lives. Twenty percent of the study sample encountered basic problems and 28 % social problems (Table 1). Only 11 % of the respondents had basic problems as well as social problems. Sexual (14 %) or financial (12 %) problems were mentioned most often.

**Table 1:** Everyday problems of people with chronic illness

Everyday problems	Number	Percent
No problems	1087	63 %
Basic problems	351	20 %
Finances	208	12 %
Housing	84	5 %
Work	147	9 %
Social problems	485	28 %
Leisure pursuit	149	9 %
Partner	129	8 %
Children	89	8 %
Friends	138	8 %
Sex	238	14 %

Both types of everyday problems were negatively associated with age and perceived health (Table 2). These associations indicate that the older people are, or the higher they rate their general health, the less likely it is that they encounter basic and social problems in their everyday life. In addition, respondents who experienced (mild, moderate or severe) physical limitations had significantly higher odds of having everyday problems than people who did not experience physical limitations, except for respondents with severe physical limitations regarding having basic problems.

**Table 2:** Multivariate logistic regression analyses testing the relation between everyday problems and socio-demographic, illness and health status characteristics (*n* = 1501)

	Everyday problems	
	Basic problems	Social problems
	OR	OR
Socio-demographic characteristics		
Age	.95**	.97**
Female	.81	.78
Educational level		
Low	Ref.	Ref.
Intermediate	1.17	1.49**
High	.90	1.38
Illness characteristics		
Type of disease(s)		
Cardiovascular disease	.81	.90
Asthma	1.08	1.12
COPD	.98	1.07
Musculoskeletal disorder	.69	1.05
Cancer	.79	1.24
Diabetes mellitus	1.00	1.00
Neurological disease	.90	1.05
Gastrointestinal disease	.85	.74
Other chronic disease	.81	1.01
Multimorbidity	1.04	.99
Health status characteristics		
Perceived general health	.98**	.98**
Physical limitations		
No limitations	Ref.	Ref.
Slight limitations	2.05**	1.98**
Moderate limitations	2.31**	2.53**
Severe limitations	1.35	2.27**

*Significant at *p* < .05 **Significant at *p* < .01

### Everyday problems and self-management

Chronically ill people with basic or social problems reported lower levels of self-management than people who did not have everyday problems (Tables 3 and 4). Adjusting for covariates reduced the differences between the two groups, although some differences remained significant. Regarding basic problems, there was no difference in symptom management (hypothesis 1) and active involvement in treatment (hypothesis 2) between people who have basic problems and people who do not have basic problems. However, people who experienced basic problems were less actively coping with the consequences of their illness than people who did not have those problems (hypothesis 3). Regarding social problems, people who experienced social problems had a lower level of symptom management (hypothesis 1) and were also less active in coping (hypothesis 3). There were no differences between people who had social problems and those who did not regarding symptom management (hypothesis 2).

**Table 3:** Mean level of self-management comparison between chronically ill people who have no basic problems and chronically ill people who have basic problems, unadjusted means versus PSM adjusted estimates^a^

	Unadjusted means (*n*)		Model-based (adjusted) estimates		
	No basic problems	Basic problems	Mean difference	95 % CI	*p*-values
Self-management					
Symptom management	2.97 (1174)	2.91 (301)	.01	−.27–.28	n.s.
Active involvement	3.37 (1182)	3.21 (299)	−.10	−.23–.03	n.s.
Coping	2.78 (1183)	2.15 (303)	−.29	−.44– −.15	0.000

^a^Models are adjusted for age, sex, education, comorbidity, perceived general health and physical limitations

**Table 4:** Mean level of self-management comparison between chronically ill people who have no social problems and chronically ill people who have social problems, unadjusted means versus PSM adjusted estimates^a^

	Unadjusted means (*n*)		Model-based (adjusted) estimates		
	No social problems	Social problems	Mean difference	95 % CI	*p*-values
Self-management					
Symptom management	3.01 (1053)	2.83 (422)	−.16	−.31– −.02	0.026
Active involvement	3.36 (1059)	3.29 (422)	−.06	−.16–.05	n.s.
Coping	2.86 (1062)	2.13 (424)	−.37	−.48– −.25	0.000

^a^Models are adjusted for age, sex, education, comorbidity, perceived general health and physical limitations

The sensitivity analyses show similar results (Appendixes 1 and 2). We also found that having both basic and social problems had an interaction effect on coping (Appendix 3). This indicates that the negative association of having basic or social problems with the level of coping was stronger when people had both basic and social problems. An interaction effect was not found for the other two domains of self-management.

## Discussion

Being chronically ill is not a ‘one moment stressful life event’, but a continuous process of balancing the demands of the illness and the demands of everyday life. The basic assumption of this study was that performing self-management activities is more complicated when people have basic and social problems in their everyday life. This study shows that having everyday problems is indeed related to lower levels of self-management. The effect of everyday problems on self-management depends on the type of problems people with chronic illness encounter on a daily basis, as well as on the type of self-management at stake.

One third of the people with chronic illness encounters basic or social problems in their everyday life. Interestingly, having everyday problems is negatively associated with age. Studies show that older adults’ lives are less stressful compared to the lives of middle-aged adults, as they report fewer daily stressors and their routines are less disrupted by stressors [27–29]. Furthermore, people with chronic illness are more likely to experience everyday problems when they have physical limitations and when they perceive their health as poor. This is not surprising as some everyday problems might be a direct consequence of having a chronic illness. For instance, people might have problems with their work because of a limited amount of energy due to the chronic illness.

In line with our first hypothesis, the level of recognition and management of symptoms was lower when people have social problems in their daily life. However, in contrast to what we expected, people who had basic problems, such as financial, housing or work problems, did not display a lower level of symptom management than people who did not have those problems. The reason why only social problems were associated with symptom management may be related to the nature of social problems. Having social problems could be a sign of a lack of social support. Studies have shown that good social support has a positive effect on self-management [30].

In contrast with our second hypothesis, having basic or social problems in everyday life did not have an (small) effect on the level of active involvement in the treatment, such as adhering to treatment regimens, visiting healthcare providers and participating in decision-making. Almost all respondents scored really high on this aspect of self-management, which might indicated that we only measured a basic level of active involvement. In addition, active involvement in treatment will be established in a medical context in close collaboration with healthcare professionals. Therefore, active involvement will not only depend on the patient, but also on the healthcare professional. This probably more easily activates a frame where managing the chronic illness in this respects gets priority.

Finally, we found that having basic and social problems was related to less coping with the consequences of having a chronic illness, such as dealing with the effects of being chronically ill on physical, emotional and social wellbeing. In line with our third hypothesis, coping (from all three self-management dimensions we assessed) appeared to be most affected by having everyday problems. In addition to their negative main effects, having both basic and social problems accumulated in an even lower level of coping. This is an important finding as it might explain why a person with a chronic illness is not able to accept the chronic illness or make the desired lifestyle changes.

### Strengths, limitations and future research

A strength of this study is the use of data from a nationwide representative sample of people with chronic illness. This provides unique insights into the perceptions of people with chronic illnesses. In addition, this study is one of the first to examine the effect of everyday problems on the level of self-management of people with chronic illness. We did so by using PSM.

A limitation of this study is that its cross-sectional design means we cannot determine causality; PSM is only an approximation. We aimed to study whether and how everyday problems of people with chronic illness interfere with their self-management, but we cannot reject the reversed effect, namely that poor self-management of a chronic illness results in experiencing (more) everyday problems. We have tried to minimise the problem by using PSM. Another limitation is formed by the fact that we lacked information about the severity of the problems. People could have, for instance, minor financial problems (not being able to go on holiday) or major financial problems (struggling to get by each month). Despite this lack of information about the severity of the problems, we did find a negative association with the level of self-management. This negative association might have been even stronger if we could have included the severity of the problems people with chronic illness encounter. Finally, there are some other socio-demographic characteristics, next to age, gender and highest level of education, that could have influenced both self-management behaviour and having everday problems, such as family arrangements and income. Further research should take those characterisics also into account.

Longitudinal studies are needed to establish whether and in what way everyday problems result in lower levels of self-management. Further research should examine more precisely which types of everyday problems have an effect on self-management and whether combinations of certain problems have an accumulating effect on self-management. Also, the theoretical idea that people set priorities in which problems to address, given their limited resources, and that these priorities are influenced by how they see their personal situation, needs more research.

## Conclusion

It was already known that being chronically ill can be disruptive to people’s daily life. However, this study shows that this effect might work both ways and that everyday problems of people with chronic illness interfere with their self-management. The effect of these problems on self-management depends on the type of problems people with chronic illness encounter on a daily basis as well as on the type of self-management at stake. Healthcare providers should therefore actively address the individual (social) circumstances of people with chronic illness and the broader context in which self-management of chronically ill people takes place. Seeing self-management as part of people’s individual life context might help to understand the difficulties people with chronic illness might have with self-management and, in many cases, to subsequently resolve them.

### Authors’ statement of adherence to ethical standards

All procedures, including the informed consent process, were conducted in accordance with the Helsinki Declaration of 1975, as revised in 2000. According to the Dutch Medical Research Involving Human Subjects Act, this study does not require ethics approval.

## Appendix 1

**Table 5:** Linear regression analyses testing the effect of basic problems on self-management, controlling for socio-demographic, illness and health status characteristics

	Self-management		
	Symptom management	Active involvement	Coping
	(*n* = 1475)	(*n* = 1481)	(*n* = 1486)
	Coef.	Coef.	Coef.
Everyday problems			
Basic problems	−.05	−.11*	−.38**
Covariates			
Age	−.00	.01**	.01**
Female	.19**	.05	−.02
Educational level			
Low	Ref.	Ref.	Ref.
Intermediate	.02	.08	.06
High	−.02	.06	−.00
Multimorbidity	.06	.07	−.01
Perceived general health	.00	.00	.02**
Physical limitations			
No limitations	Ref.	Ref.	Ref.
Slight limitations	−.08	−.01	−.26**
Moderate limitations	−.14	−.01	−.44**
Severe limitations	−.16	.04	−.74**

*Significant at *p* < .05 **Significant at *p* < .01

## Appendix 2

**Table 6:** Linear regression analyses testing the effect of social problems on self-management, controlling for socio-demographic, illness and health status characteristics

	Self-management		
	Symptom management	Active involvement	Coping
	(*n* = 1475)	(*n* = 1481)	(*n* = 1486)
	Coef.	Coef.	Coef.
Everyday problems			
Social problems	−.17**	−.04	−.42**
Covariates			
Age	−.00	.01**	.01**
Female	.18**	.06	−.02
Educational level			
Low	Ref.	Ref.	Ref.
Intermediate	.03	.08	.08
High	−.01	.06	.03
Multimorbidity	.07	.07	−.00
Perceived general health	−.00	.00	.02**
Physical limitations			
No limitations	Ref.	Ref.	Ref.
Slight limitations	−.07	−.02	−.24**
Moderate limitations	−.12	−.02	−.41**
Severe limitations	−.13	.05	−.69**

*Significant at *p* < .05 **Significant at *p* < .01

## Appendix 3

**Table 7:** Linear regression analyses testing the interaction effect of basic and social problems on self-management, controlling for socio-demographic, illness and health status characteristics

	Self-management		
	Symptom management	Active involvement	Coping
	(*n* = 1475)	(*n* = 1481)	(*n* = 1486)
	Coef.	Coef.	Coef.
Everyday problems			
Basic problems	.00	−.14*	−.17*
Social problems	−.16*	−.03	−.29**
Interaction effect	−.02	.07	−.26*
Covariates			
Age	−.00	.01**	.01*
Female	.18**	.05	−.03
Educational level			
Low	Ref.	Ref.	Ref.
Intermediate	.03	.08	.09
High	−.01	.06	.02
Multimorbidity	.07	.07	−.01
Perceived general health	−.00	.00	.02**
Physical limitations			
No limitations	Ref.	Ref.	Ref.
Slight limitations	−.07	−.01	−.23**
Moderate limitations	−.12	−.01	−.39**
Severe limitations	−.13	.05	−.69**

*Significant at *p* < .05 **Significant at *p* < .01

## Abbreviations

NPCD: National Panel of people with Chronic illness or Disability
GP: General practitioner
PSM: Propensity score matching
